# High Potential of *Pichia kluyveri* and Other *Pichia* Species in Wine Technology

**DOI:** 10.3390/ijms22031196

**Published:** 2021-01-26

**Authors:** Javier Vicente, Fernando Calderón, Antonio Santos, Domingo Marquina, Santiago Benito

**Affiliations:** 1 Unit of Microbiology, Genetics, Physiology and Microbiology Department, Biology Faculty, Complutense University of Madrid, Ciudad Universitaria S/N, 28040 Madrid, Spain; javievic@ucm.es (J.V.); ansantos@ucm.es (A.S.); dommarq@bio.ucm.es (D.M.); 2 Department of Chemistry and Food Technology, Polytechnic University of Madrid, Ciudad Universitaria S/N, 28040 Madrid, Spain; fernando.calderon@upm.es

**Keywords:** *Pichia kluyveri*, thiols, higher alcohols, esters, fatty acids, wine, *P. anomala*, *P. fermentans*, *P. guilliermondii*, *P. kudriavzevii*, *P. membranifaciens*

## Abstract

The surfaces of grapes are covered by different yeast species that are important in the first stages of the fermentation process. In recent years, non-*Saccharomyces* yeasts such as *Torulaspora delbrueckii*, *Lachancea thermotolerans*, *Metschnikowia pulcherrima*, and *Pichia kluyveri* have become popular with regard to winemaking and improved wine quality. For that reason, several manufacturers started to offer commercially available strains of these non-*Saccharomyces* species. *P. kluyveri* stands out, mainly due to its contribution to wine aroma, glycerol, ethanol yield, and killer factor. The metabolism of the yeast allows it to increase volatile molecules such as esters and varietal thiols (aroma-active compounds), which increase the quality of specific varietal wines or neutral ones. It is considered a low- or non-fermentative yeast, so subsequent inoculation of a more fermentative yeast such as *Saccharomyces cerevisiae* is indispensable to achieve a proper fermented alcohol. The impact of *P. kluyveri* is not limited to the grape wine industry; it has also been successfully employed in beer, cider, durian, and tequila fermentation, among others, acting as a promising tool in those fermentation processes. Although no *Pichia* species other than *P. kluyveri* is available in the regular market, several recent scientific studies show interesting improvements in some wine quality parameters such as aroma, polysaccharides, acid management, and color stability. This could motivate yeast manufacturers to develop products based on those species in the near future.

## 1. Introduction

Usually, indigenous non-*Saccharomyces* yeasts are present in grape musts in greater numbers than *Saccharomyces cerevisiae*, the yeast that dominates wine fermentation when the ethanol content gets over 4%. That is why must fermentation is not naturally a single-species process. Grapes contain diverse yeast species that will define the final fermented product [1]. When wine science began its development, non-*Saccharomyces* yeasts were often seen as pernicious, associated with microbial-related problems due to their regular presence in spoiled wines. Currently, science is clarifying the role they perform, and several non-*Saccharomyces* yeasts are considered to have a positive role in the wine industry [2,3,4].

Different non-*Saccharomyces* yeast species have typically been evaluated for their influence on the overall quality of wine (Table 1) [5,6]. Some of these attributes are highly strain dependent, and there must be a proper selection process in order to select the most appropriate strains to improve wine quality at the industrial level.

The growing interest in *P. kluyveri* is reflected in the number of scientific publications regarding this species. According to the PubMed^®^ database, over a period of 10 years (2009–2019), 33 publications were related to *P. kluyveri* and wine, of which 14 were in the last two years (2018 and 2019). Despite the growing interest, it is still far from the interest shown for other wine-related yeast species. In the same period, 1503 works on *S. cerevisiae* and wine, 114 on *Metschnkowia* spp., 116 on *T. delbrueckii*, and 75 on *L. thermotolerans* were published.

Different *Pichia* species that have been found in must fermentations and are considered to be wine-related are included in the *non*-*Saccharomyces* group: *P. fermentans*, *P. membranifaciens*, *P. occidentalis*, *P. terricola*, *P. manshurica*, *P. kudriavzevii*, and *P. kluyveri*. The frequency of isolation of *Pichia* species from grapes is lower than that of *S. cerevisiae* (28%) and other non-*Saccharomyces* such as *Hanseniaspora uvarum* (44%). The frequency varies from 0.12% for *P. occidentalis* up to 4.7% for *P. anomala*. Other reported *Pichia* species usually isolated from grapes are *P. manshurica* (2.81%), *P. menbranifaciens* (0.98%), and *P. kudriavzevii* (0.85%) [14]. Those lower frequencies justify the lack of commercial strains compared to other species, making it difficult to make a proper selection.

Among the wine-related *Pichia* species, *P. kluyveri* is the most studied and is the only one commercially available in the yeast market currently. *P. kluyveri* is characterized by its ability to improve the composition of aromatic compounds such as thiols, terpenes, and fruity esters. Currently, there are only two commercial starters based on *P. kluyveri*: WLP605 (Vintner’s Harvest^®^, Yakima, WA, USA), which is advertised as increasing rose petal and floral aromas, contributing to improve the overall bouquet of wine, and FROOTZEN^®^ (Hansen^®^, Hoersholm, Denmark), which is advertised as increasing varietal and thiolic aromas [4,15]. Both are indicated for use in sequential fermentation, first with *P. kluyveri*, and 48 h later with a *S. cerevisiae* strain, which will properly end the alcoholic fermentation.

*Pichia* species show multilateral buds for asexual reproduction, whereas sexual reproduction is characterized by unconjugated asci; the conjugation occurs between a parent cell and its bud or between independent cells. Asci may be persistent or deliquescent, with usually one to four and more rarely five to eight ascospores. The ascospores are rough or smooth and spherical to hat-shaped, and sometimes they present equatorial or subequatorial ledges. The cell shape is spherical to ovoid and occasionally may appear as pseudohyphae. *Pichia* spp. can ferment glucose but rarely other sugar molecules. The genus assimilates some sugars and is not able to assimilate nitrate as a nitrogen nutrient. The genus produces coenzyme Q-7 [16]. The last genus revision described 20 accepted species, among which *Pichia membranifaciens* is considered the type species [16]; only a few of them are considered positive in winemaking.

**Figure 1:**
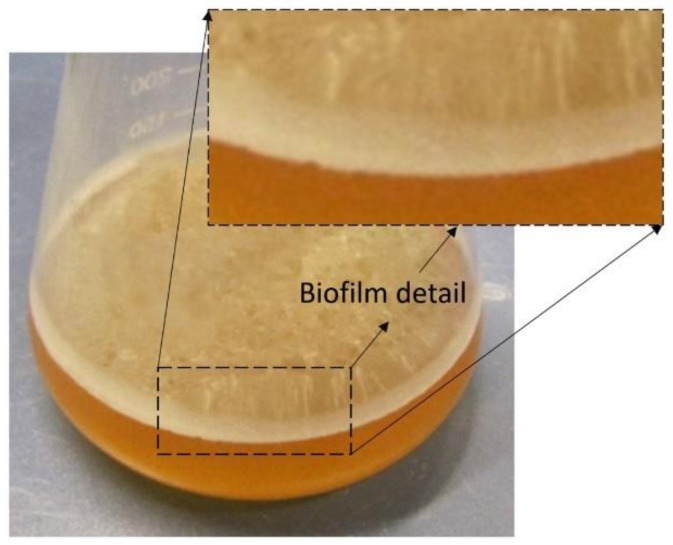
Film produced by *Pichia kluyveri* over grape must at the beginning of alcoholic fermentation.

As far as *P. kluyveri* is concerned, the cells are slightly ovoid and about 2–10 µm, and it is very difficult to distinguish their shape from the shape of *S. cerevisiae* or *S. ellipsoideus* cells. Its ability to produce a film during its development in must is very characteristic and allows us to easily distinguish the species among other yeasts (Figure 1). The ability to distinguish this film formation is very useful at the industrial scale to quickly evaluate implantation success when using commercial products that contain *P. kluyveri*. This species is able to produce pseudohyphae in plate cultures but not in liquid fermentation. It can also produce hat-shaped ascospores. The species only ferments glucose and shows growth in liquid media containing glucose, ethanol, or glycerol. Like other *Pichia* species, *P. kluyveri* resists high osmotic pressure, presenting optimal growth in 10% NaCl or 5% glucose [16]. It has been usually isolated from rooted fruit and green parts of plants, being widely distributed in all type of ecosystems [17].

**Figure 2:**
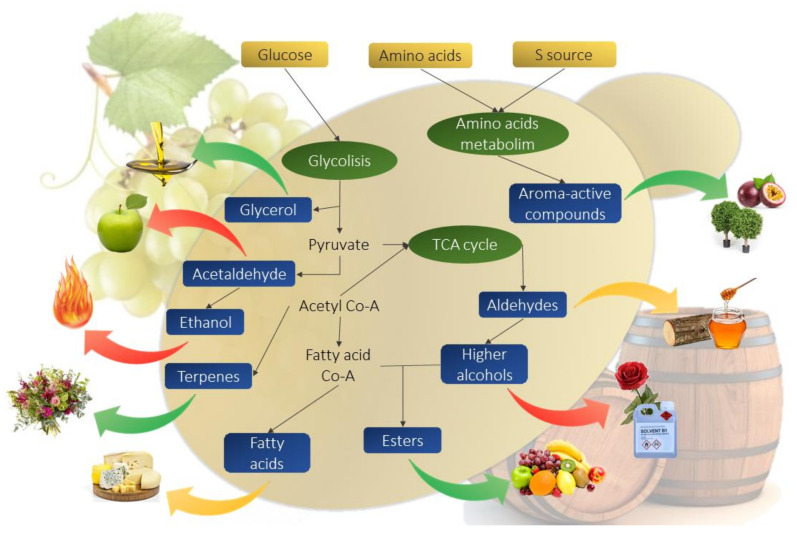
Diagram of main metabolic processes under fermentation conditions in *P. kluyveri*. Arrow color indicates variation compared to *S. cerevisiae* metabolism (red: decreased; yellow: no difference; green: increased). Adapted from [18].

This review gathered all available information related to *P. kluyveri* and its influence on must fermentation considering that wine is a product obtained due to yeast metabolism. Despite that *P. kluyveri* presents a more oxidative metabolism than *S. cerevisiae,* Figure 2 shows the most common metabolic routes in *P. kluyveri* under fermentation conditions and its sensorial influence on wine. Additionally, this review also examined other *Pichia* species that are attracting increasing oenological interest.

## 2. *P. kluyveri* Impact on Different Wine Quality Parameters

### 2.1. Ethanol

*P. kluyveri* is only able to ferment up to 4–5% (*v*/*v*) in ethanol, consuming only glucose and leaving fructose [19]. This fermentation capacity is insufficient to produce regular wines or sparkling base wines but is enough to produce other beverages such as beer of about 3.2% (*v*/*v*) [20] or tequila base [21].

The ethanol yield of *P. kluyveri* is 22% lower than that of *S. cerevisiae*, producing 0.36 g of ethanol per gram of sugar. Most *Pichia* species have lower yields than *P. kluyveri*, such as *P. fermentans* (0.04 g), *P. membranifaciens* (0.08 g), *P. terrricola* (0.19 g), and *P. kudriavzevii* (0.33 g). However, some *Pichia* species have been shown to have a higher ethanol yield; for example, *P. holstii* yields around 0.43 g of ethanol per gram of sugar [22].

Since *P. kluyveri* is unable to ferment fructose and consume the full amount of glucose present in grape juice, it must be combined with fermentative yeast such as *S. cerevisiae* to completely ferment sugars and achieve the desired quality parameters. Sequential fermentation involving *P. kluyveri* and *S. cerevisiae* resulted in lower final ethanol content than *S. cerevisiae* controls. The difference increased in fermentations when *P. kluyveri* was present for a longer time during the winemaking process. Sequential inoculation at 48 h resulted in a lower ethanol content of 0.16% (*v*/*v*) [23], while another sequential inoculation at 96 h resulted in 0.25% (*v*/*v*) [24].

The ethanol reduction is due to the oxidative metabolism of non-*Saccharomyces* species that consume glucose without ethanol formation [25]. The sugar that is not converted into ethanol is transformed into other compounds, such as glycerol or acids [23]. Among those species, *P. kluyveri* is the second most efficient among 23 studied species, after *M. pulcherrima*. When it was employed in sequential fermentation, the ethanol content was reduced between 3 and 22% [22,25,26].

With regard to the fermentation kinetics, coinoculation of *P. kluyveri* and *S. cerevisiae* in a 9:1 ratio presumed a final delay of 3 days in alcoholic fermentation compared to the *S. cerevisiae* control [27]. In that study, *P. kluyveri* cells were detected during the first 9 days in an alcoholic fermentation that lasted for 23 days at 14 °C. The sequential inoculation strategy allowed the detection of *P. kluyveri* until 6 days after *S. cerevisiae* inoculation, which occurred 8 days later than the *P. kluyveri* inoculation [28]. However, other studies reported a fast to immediate decrease after *S. cerevisiae* inoculation [23], which reinforces the importance of selecting a compatible *S. cerevisiae* partner that allows the virtues of *P. kluyveri* to be increased during alcoholic fermentation.

### 2.2. Glycerol

Glycerol concentration is higher in sequential fermentation involving *P. kluyveri* than in *S. cerevisiae* controls, and the effect increases when *P. kluyveri* ferments longer. A sequential inoculation of 48 h resulted in an increase in glycerol of 0.33 g/L [23], while another inoculation of 96 h resulted in a higher increase of 1.3 g/L [24]. Other studies reported a decrease of about 48% in coinoculation [27]. This difference could be explained by possible strain variability similar to that reported for other non-*Saccharomyces* species. Although some studies reported positive significant increases in final glycerol concentration related to *P. kluyveri* performance, other non-*Saccharomyces* such as *C. zemplinina* are much more efficient for this purpose, able to produce a final glycerol concentration up to 15 g/L [5].

### 2.3. Organic Acids

*P. kluyveri* does not notably influence wine organic acids as other specific non-*Saccharomyces* do. It is reported to slightly consume malic acid in a concentration of about 0.1 g/L [24]. However, that is not enough to significantly influence the pH or achieve malic acid microbiological stability [29]. All studies involving *P. kluyveri* have reported nonsignificant statistical differences in acetic acid production between *P. kluyveri* sequential fermentation and *S. cerevisiae* control [23,24,27].

One study reported increments of some acids derived from the tricarboxylic acid cycle under sequential fermentation involving *P. kluyveri*: α-ketoglutaric acid (24%), oxalic acid (50%), and succinic acid (300%) [30]. In this study, the control was a *T. delbrueckii* strain and the fermentative product was durian wine. There are no available data yet for grape wine compared to *S. cerevisiae* control, so further studies must be performed on this topic, as similar results could occur with grape juice fermentation. Succinic acid concentration in wine usually varies from 0.5 to 1 g/L [31], so final concentrations up to 5 g/L by sequential inoculations reported for *P. kluyveri* could be an interesting alternative to wine acidification. As those concentrations are over the average value for wine, it is probable that they significantly influence its sensorial properties. While citric, L-lactic, L-malic, and L-tartaric acids are described as sour and astringent from a sensorial point of view, succinic acid is described as sour, salty, and bitter. However, the study does not include a sensory analysis to corroborate the possible influence of succinic acid on the final flavor [30].

### 2.4. Aroma Compounds

*P. kluyveri* species showed a remarkable ability to release **3**-sulfanylhexan-**1**-ol acetate (3_SHA) compared to other *Saccharomyces* and non-*Saccharomyces* species [27]. **3**-SHA is a pleasant volatile molecule that produces desired aromas in wine described as passionfruit or box tree. A study reported that sequential fermentation involving *P. kluyveri* and *S. cerevisiae* reached notably higher final concentrations of **3**-SHA than the control fermented only by *S. cerevisiae*. The increases varied from 10 to 72% depending on the initial inoculation ratio between *S. cerevisiae* and *P. kluyveri*. The optimum reported initial ratio was 1:9 and the final **3**-SHA concentration varied from 55 to 72% higher than the *S. cerevisiae* control depending on the *P. kluyveri* strain that performed the fermentation. The increase of **3**-sulfanylhexan-**1**-ol (**3**-SH) was about 40% for the 1:9 ratio inoculation. Statistically significant differences in thiol release by *P. kluyveri* were reported to depend on the *S. cerevisiae* strain employed to properly end the alcoholic fermentation [27]. Those results suggest that the *S. cerevisiae* partner must be carefully selected to optimize the final total thiol concentration released during alcoholic fermentation when working together with the selected *P. kluyveri* strain. As significant strain variability regarding thiol release is reported for *P. kluyveri* [27], selecting yeast strains with high β-lyase activity, similar to other non-*Saccharomyces* species such as *T. delbrueckii* or *M. pulcherrima*, could optimize *P. kluyveri* thiol release activity [32].

*P. kluyveri* reduces the content of total higher alcohols under sequential fermentation by about 15% [23], with each higher alcohol affected in a different range (e.g., hexanol, –50%; **2**-phenyl-ethanol, −20%; and butanol, −20%). Other studies reported the same results, with variations in different ranges [33] ((Z)-4-decen-**1**-ol, −9%; (E)-4-decen-**1**-ol, −8%; **1**-decanol, −4%; **1**-hexanol, −28%; **1**-nonanol, −12%; **2**-hepten-**1**-ol, −32%; **2**-methyl-**3**-buten-**1**,**2**-diol, −14%; **3**-octanol, −11%; **5**-nonanol, −20%; and cyclooctanemethanol,α,α,-dimethyl, −12%). A similar effect was previously observed in other non-*Saccharomyces* such as *Torulaspora*, *Lacchancea*, and *Metschnikowia* [5]. Other studies observed an increase in higher alcohols of around 25% and great variation among them (e.g., hexanol, +12% and **2**-phenyl-ethanol, +25%) [24]. The latest biotechnology techniques for producing varietal wines tend to reduce as much as possible the production of higher alcohols to values below 350 mg/L because they mask the varietal aroma compounds [34]. The final total higher alcohol concentration reported for sequential fermentation between *P. kluyveri* and *S. cerevisiae* was always below 350 mg/L, varying from 176 [23] to 254 mg/L [24].

Different studies report a higher production of total esters for sequential fermentation involving *P. kluyveri* than *S. cerevisiae* controls. A study on the presence of different enzymatic activities of oenological impact [35] reported that all studied strains of *P. kluyveri* presented esterase activity, which catalyzes the formation of esters. The highest increase was 25% for **2**-phenyl ethyl acetate [23] and 50% [24] for a longer sequential inoculation. Another study reported further increases up to 60% in red wine [33]. The compound **2**-phenyl-ethyl acetate is a desirable aromatic compound that increases the perception of aromas such as rose or floral when it appears in concentrations over 0.25 mg/L [34]. The yeast strains employed to ferment neutral varieties such as Airen and Ugni blanc, which did not possess high levels of molecules such as terpenes or thiols, are selected to enhance the final fruity ester concentration. On the contrary, yeast strains employed to ferment varieties with strong varietal characteristics such as Verdejo, sauvignon blanc, and Muscat are selected to produce lower levels of esters in order to not mask the varietal aromas [34].

Several studies reported no effect of *P. kluyveri* on the final concentration of total terpenes compared to *S. cerevisiae* control [23,24,33]. This is mainly because β-glucosidase activity is reported to be not common in *P. kluyveri* strains [35]. Other factors that could affect this phenomenon are the initial sterilization of the grape juice and the performance of varieties with low terpene content. Indeed, differences in specific terpenes are reported. It was reported that *P. kluyveri* sequential fermentation had higher levels of linalool oxide and hotrienol by about double and 40%, respectively, while the concentration of nerol was lower by about 10% [23].

Total fatty acid content was not influenced by *P. kluyveri* [23] or even decreased, and decanoic acid was the most affected, with decreased concentration by around 18% [24]. These results agree with a report showing the absence of lipase enzymes in *P. kluyveri* species [35]. Specific fatty acids such as isovaleric acid stood out due to an increase of around 25% compared to *S. cerevisiae* control [24]. The production of isovaleric acid should be taken into account for strain selection, as concentrations over 50 mg/L produce undesirable aromas such as rancid cheese [34]. This phenomenon has been previously reported for other non-*Saccharomyces* species such as *T. delbrueckii* [8].

One study reported lower production of acetaldehyde by nearly 40% compared to *S. cerevisiae* control [23], although the final values were far below the olfactory threshold of 125 mg/L and related to undesirable oxidative descriptors. This additional effect could increase the impact of other varietal aroma compounds such as thiols and terpenes, as they are less masked for this significant aromatic compound that produces oxidative aromas. Table 2 summarizes the main aroma compounds influenced by *P. kluyveri*.

Some studies have reported some off-odor compounds in sequential fermentation with *P. kluyveri* compared to *S. cerevisiae* control. Some studies reported an increase in fatty acid content, such as **3**-methyl-butanoic acid (isovaleric acid) and phenylamine, which are linked to undesirable aromas, such as cheese, sweaty feet, or off-putting sourness [24,28]. *P. kluyveri* has been reported to produce higher levels of H_2_S by about 50% in sequential fermentation, although the final value was below the fault threshold [24].

### 2.5. Amino Acids

Only one study reported the final content of amino acids in sequential fermentation with *P. kluyveri* compared to conventional fermentation by *S. cerevisiae* [23]. *P. kluyveri* sequential fermentation produced about 10% less final threonine than *S. cerevisiae* pure fermentation. There were no differences in ornithine, while the concentration of other studied amino acids was always higher for *P. kluyveri* sequential fermentation. Slight increases varying from 5% to 15% were reported for asparagine, alanine, leucine, and glycine, while higher increases of about 50% were reported for aspartic acid, arginine, phenylalanine, isoleucine, lysine, serine, and tyrosine. Increased final amino acid content is usually related to less nutrient nitrogen consumption or higher cellular release.

There are no scientific reports on *P. kluyveri* as a biogenic amine producer [37]. The increased final concentration of tyrosine and lysine could evolve to tyramine and cadaverine if they were decarboxylated by lactic bacteria during malolactic fermentation or an undesirable contamination process [5]. The 50% higher final concentration of phenylalanine than *S. cerevisiae* control could explain the higher isovaleric acid production observed in other studies [23,28]. Isovaleric acid can be produced from other amino acids due to aromatic-L-amino-acid decarboxylase enzyme [28].

## 3. Sensory Impact in Regular Wine Fermentation

Only one scientific study performed a sensorial evaluation of *P. kluyveri* fermentation, analyzing 17 parameters. The study reported sequential fermentation involving *P. kluyveri* to show a better overall impression by about 30% compared to *S. cerevisiae* control [23]. The better aromatic quality was related to a higher fruity character [27], mainly peach, apricot, citrus, and grapefruit, which could be related to an increase in thiol release (mainly **3**-SHA) during the alcoholic fermentation. That study also reports higher Riesling grape varietal typicity commonly related to those descriptors. Other studies showed no increases from a sensorial point of view of those descriptors compared to *S. cerevisiae* control [28]. The differences can be related to the absence of thiol precursors or to yeast strain variability.

Although **3**-SH appears in higher concentrations than **3**-SHA, the corresponding perception threshold is 60 and 4 ng/L. For that reason, **3**-SHA has a 15 times higher impact on wine than **3**-SH, justifying its higher importance. The specific aroma descriptors of **3**-SHA are passionfruit, box tree, and boxwood, which describe the typicity of some of the best sauvignon blanc in the world [34].

## 4. Killer Factor and Its Influence on Wine Ecology

Wine spoilage associated with different non-*Saccharomyces* strains is a major concern for winemakers. It is therefore important to develop a reliable and effective procedure to inhibit the presence of such yeasts. To achieve this purpose, sulfur dioxide (SO_2_) is widely used, but the antimicrobial effect of this compound is affected by the wine composition, and it can cause allergic reactions in wine consumers. Alternatives for biologically controlling its abundance are therefore actively sought [38]. This section of the review is focused on describing the killer toxins produced by *P. kluyveri*, which are antimicrobial compounds that have shown potential for inhibiting different spoilage yeasts in wine.

Several yeast genera and species can produce and secrete toxic proteins that inhibit many sensitive filamentous fungi and yeasts. The ability to produce killer toxins is strain-dependent, usually related to infection by virus-like particles of the Totiviridae family. However, in the *Pichia* genus, several toxins have been described to be either associated with cytoplasmic genetic elements, such as dsDNA virus-like elements, or chromosomally encoded [39].

Pichia is one of the most prolific yeast genera in the production of different kinds of killer toxins. *P. acaciae, P. inositovora, P. anomala, P. kluyveri*, and *P. membranifaciens*, which are widespread in nature and in wine-related environments, have been identified as killer yeasts and several of their toxins have been characterized. Most of these toxins are outside the scope of the present review, but their characteristics and potential applications have been previously reviewed [39].

As far as *P. kluyveri* is concerned, two killer toxins have been described. The first killer toxin described for *P. kluyveri* 1002 is a 19.0 kDa glycoprotein that is chromosomally inherited, showing its mayor activity in pH from 3.8 to 4.0. Its mechanism of action is based on membrane permeabilization [40]. The second killer toxin, Pkkp, has a greater molecular mass (54.0 kDa) and higher optimum pH value (4.0–4.5) and its primary receptor is in the cell wall; its mechanism of action has not been elucidated [41].

The first killer toxin of *P. kluyveri* has an antimycotic effect on sensitive cells. It is produced in the exponential growth phase and allows an advantage in must fermentation, although it is associated with a cost. The cost comes from a delay in reaching the exponential growth phase compared to the non-killer phenotype [42].

Sulfur dioxide, the most popular antimicrobial control agent in winemaking, is starting to come under legal restrictions; consequently, newly developed alternatives are starting to be used [38]. The utilization of killer yeasts such as *P. kluyveri* has been proposed as an interesting alternative to biologically controlling the initial level of undesirable microorganisms. For example, a Pkkp-producer strain has been demonstrated to be active against a wide variety of food and beverage spoilage yeasts such as *Brettanomyces/Dekkera bruxellensis* [41]. However, this killer toxin can inhibit *S. cerevisiae* in beverages, so as a biocontrol agent in winemaking, it should be used carefully [41]. It must be considered that when killer yeasts are used as antimicrobial agents in fermentations conducted using *S. cerevisiae*, a resistant *S. cerevisiae* strain must be used in order to ensure that the alcoholic fermentation in sequential inoculations is finished [40]. *P. kluyveri* has been found to be a killer species against low fermentative or oxidative yeasts such as *Candida bodinii, C. patagonica*, and *Geotrichum silvicol*, which develop a surface biota in incompletely filled barrels or vats [43].

Other *Pichia* toxins, such as *P. membranifaciens* killer toxin (PMKT), show synergistic interaction when used in combination with food antimicrobials such as potassium metabisulphite [44]. This characteristic has been proposed to reduce preservative concentration in foods and beverages; unfortunately, this seems to be an unusual characteristic because it is not observed for Pkkp [41].

## 5. Influence of Other *Pichia* Species on Winemaking

### 5.1. Pichia Guilliermondii

Although most applications of *Pichia* species in winemaking are related to improvements in aroma composition (Table 3), *P. guilliermondii* is applied to improve wine color properties [45]. *P. guilliermondii* is the yeast species that shows the most hydroxycinnamate decarboxylase enzymatic activity. Hydroxycinnamate decarboxylase produces pyranoanthocyanin adducts, which are the most stable colored compounds in wine chemistry [46]. These compounds are responsible for maintaining the color of wine during long aging periods. In other yeast genera, such as *Saccharomyces*, enzymatic activity is a strain-dependent characteristic. The maximum enzymatic activity reported for *Saccharomyces* is about 15% [45], while the maximum reported for *P. guilliermondii* is up to 90%. These differences in enzymatic activity allow *P. guilliermondii* to produce higher content of pyranoanthocyanin adducts, from 6 to 10 times higher than *S. cerevisiae* controls. Another hydroxycinnamate decarboxylase application is to capture ethylphenol precursors such as the *p*-coumaric acid [47]. This allows wines to be stabilized against ethylphenol deviations. *P. guilliermondii* has been also reported to reduce the final ethanol concentration by around 2% compared to the *S. cerevisiae* control [25]. As far as volatile compounds are concerned, esters are the most affected; *P. guilliermondii* reduces their concentration by about half under sequential inoculations with *S. cerevisiae* compared to standard control [48].

### 5.2. Pichia Kudriavzevii

*P. kudriavzevii*, the anamorph of *Candida krusei*, is widely distributed in natural substrates such us soil, fruits, and spontaneous fermentations [17]. It is an interesting tool for wine pH management, since it can significantly reduce malic acid (−40%) during alcoholic fermentation of wine and increase the pH by around 0.3 units [49,50]. Malic acid is unstable from a microbiological point of view and must be removed before bottling to avoid undesirable second fermentations in red wines. The classical method to remove malic acid to use lactic acid bacteria during malolactic fermentation. That process, especially in warm viticulture areas, can result in deviations such as a loss of color or production of harmful compounds such as biogenic amines or ethyl carbamate. For that reason, interest in studying yeasts such as *P. kudriavzevii* or *S. pombe*, which are able to remove malic acid, has increased during the last years [13,29]. As well as other *Pichia* species, *P. kudriavzevii* influences the complexity of wine aromas under sequential fermentation with *S. cerevisiae*. It increases acetate esters (by around 30%, up to 65 mg/L) and higher alcohols (by around 20%, up to 240 mg/L), and decreases fatty acids (by around 40%, up to 60 mg/L) and C6-alcohols (by around 10%, up to 1.7 mg/L) [51]. Another use of this species is to reduce the ethanol content of wines (by around 30%) [17,22].

### 5.3. Pichia fermentans

*P. fermentans* has been described as a moderate ethanol producer, reaching values of around 5% (*v*/*v*) and producing huge quantities of polysaccharides (up to 278 mg/mL, around 40%) and ethyl acetate (up to 256 mg/mL, almost 6 times higher) compared to *S. cerevisiae* control [52]. Under sequential fermentation with *S. cerevisiae*, it increased the ethanol content by around 1% (*v*/*v*) and increased most of the volatile molecules (principally thiols, acetates, and esters) in a large range [53]. *P. fermentans* has been described as low acetic acid producer in different combinations and conditions; the species does not exceed the acid levels reached in fermentation with only *S. cerevisiae*. In a two-day fermentation, it allowed a considerable increase of aromatic compounds such as acetaldehyde (70%) and glycerol (by around 1000 times) [54,55]. In a recent study using a *P. fermentans* strain as inoculum for pilot scale fermentation in 1000 L, a slight decrease in ethanol yield was observed compared to *S. cerevisiae* control (12.80% *v*/*v* compared to 13.00% *v*/*v*), and an increase in volatile acidity (20%) and volatile compounds such as acetaldehyde (10%) and **2**-methyl-**1**-propanol (53%) [56].

### 5.4. Pichia anomala

*P. anomala* is the greatest ethanol producer among *Pichia* species, producing up to 7% *v*/*v* [57]. This ability could allow the production of lower-alcohol beverages such as beer or even sparkling base wine. It is also reported to slightly increase higher alcohols, acetate, and ethyl esters, with the total content increased by around 15%, 20%, and 15%, respectively [58]. Nevertheless, its main potential could be as a biocontrol agent. It can effectively inhibit the development of spoilage molds in substrates such as malt for beer production. This antagonistic effect is due to the secretion of killer toxins; up to five killer toxins have been reported in *P. anomala* [38,59].

### 5.5. Pichia membranifaciens

*P. membranifaciens* has been reported to increase the overall quality parameters of terpenic varieties such as Muscat [60] and the content of polysaccharides (around 40%) compared to *S. cerevisiae* controls [57]. By contrast, its ethanol production is almost nil (up to 0.9% *v*/*v*) under single fermentation conditions and it increases acetic acid concentration by around four times compared to *S. cerevisiae* control [57]. With these characteristics, *P. membranifaciens* may play an important role by maintaining the microbial stability of must by producing killer toxins [38], which has been described as effective against spoilage molds such as *Botrytis cinerea* [61] and yeasts such as *Brettanomyces*/*Dekkera bruxellensis* [62].

**Table 3:** Summary of main *Pichia* species applications in winemaking.

Teleomorph	Synonym	Impact on Wine Fermentation
*Pichia kluyveri*	*Hansenula kluyveri*	Increased levels of varietal thiols (**3**-MHA) [2]
*Pichia fermentans*	*Candida lambica*	Increased levels of higher alcohols, glycerol, and polysaccharides [2]
*Pichia membranifaciens*	*Candida valida*	Increased levels of esters [4]
*Pichia terricola*	*Issatchenkia terricola*	Increased β-glucosidase activity [63]; malic acid degradation [64]
*Pichia kudriavzevii*	*Candida krusei. Issatchenkia orientalis*	Malic acid degradation [17]; increased **2**-pheyl-ethanol production [65]
*Pichia manshurica*	-	Spoilage yeast; increased volatile phenols and other off-odors [66]
*Pichia guilliermondi*	*Candida guilliermondi, Meyerozyma guilliermondi*	Increased color stability [29]
*Pichia anomala*	*Candida anomala, Hansenula anomala, Wickerhanomyces anomalus*	Increased levels of higher alcohols, acetates, and ethyl esters [2]
*Pichia pastoris*	*Komagataella pastoris, K. pseudopastoris, K. phafii*	Inhibition of protein haze formation in white wines (genetically modified strain) [67]

## 6. Proposed Selection Parameters for *P. kluyveri*

One of the main problems in selecting non-*Saccharomyces* species based on their oenological aptitude is getting a representative collection of yeast strains of a specific species. This is due to the greater presence of other non-*Saccharomyces* in grapes such as the *Hanseniaspora* genus, which represents about 50% of the yeast isolates from grapes and is the predominant genus in fermenting grape juice when ethanol goes over 4% [1]. Previous studies solved this problem by developing specific selective media such as those described for the *Schizosaccharomyces* genus [13] or incubating at higher temperatures as in the case of *L. thermotolerans* [11]. Using those methodologies, it is relatively easy to obtain a representative collection of strains without great effort or financial investment. To date, there has not been selective media described for *P. kluyveri*. So, the most efficient way to find high numbers of strains of this species is based on looking for the most appropriate substrate where this species is predominant. *P. kluyveri* is among the predominant species in olives and coffee beans, reaching an isolation frequency of about 17% and 80%, respectively [52,53], while the frequency in grapes is below 1%.

As *P. kluyveri* shows different killer factors, the selected strains cannot have an antagonist effect on the fermentative *S. cerevisiae* strain, as this second species is needed to properly end any fermentation process in combination with *P. kluyveri*. That is why any negative effect on *Saccharomyces* would not compensate for other quality improvement.

The main application of *P. kluyveri* in winemaking is based on its ability to release thiols and produce fruity or floral esters. Due to this, the first criterion of selection must be the presence of enzymes such as β-lyase and glycosidase, which enhance varietal aromas to increase the quality of terpenic varieties such as Muscat and thiol varieties such as sauvignon blanc, riesling, and Verdejo [34]. This selection process has previously been successful for other non-*Saccharomyces* species such as *T. delbrueckii* [7], *M. pulcherrima* [68], and *L. thermotolerans*. Additionally, the selection of strains able to produce high concentrations of fruity esters, such as isoamyl and octanoate acetate, or floral esters, such as **2**-penyl-ethyl acetate, would be of great interest in the making of wines from neutral grape varieties such as Airén and Ugni blanc, where varietal aromas have a low impact on the final bouquet.

The film formation of other species, such as specific *flor*-film *S. cerevisiae* strains, serves to protect the wine against oxidation in sherry winemaking [69,70,71,72]. As *P. kluyveri* is not a great fermenter, the formation of the film must be as quick as possible in order to avoid undesirable oxidative aromas and preserve the desired aromatic compounds generated by this strain that are especially sensitive to oxidation, such as thiols.

*P. kluyveri* is not considered a high fermentative yeast and always needs to be combined with a more fermentative partner such as *Saccharomyces*. We must establish the selection threshold as the maximum reported 5% (*v*/*v*) in order to facilitate fermentation as much as possible and allow *P. kluyveri* to work longer before *S. cerevisiae* inoculation [19]. Additionally, the objective ethanol yield must be about 0.36 ethanol grams per sugar gram in order to achieve wines with lower final ethanol content than those fermented only by *S. cerevisiae*. Glycerol production must also be considered in the selection process, as combined fermentations involving *P. kluyveri* are commonly described as containing higher concentrations of glycerol than *S. cerevisiae* controls. Although there are no reported data regarding SO_2_ resistance for *P. kluyveri*, most commercial non-*Saccharomyces* are especially sensitive to this additive, and one *P. kluyveri* manufacturer recommends reducing its dosage to the lowest amount possible. The selection of strains with higher resistance would increase the range of applications in winemaking. Currently, the use of *P. kluyveri* is limited to very healthy grapes that do not require higher doses of SO_2_. Despite studies reporting no significant differences in acetic acid production between fermentations involving *P. kluyveri* and *S. cerevisiae* controls, this parameter must be included in the selection process, as high strain variability, similar to that described for other non-*Saccharomyces*, could exist for this species [5].

The production of higher alcohols must be controlled to be as low as possible in order to avoid any masking effect over the varietal aromas to increase their impact as much as possible. There must be the same objective for fatty acids, especially isovaleric acid, which is described as having higher levels in fermentations involving *P. kluyveri*. Additionally, while *P. kluyveri* is described as producing less acetaldehyde than other non-*Saccharomyces*, the parameter must be verified during the selection process to avoid undesirable oxidative aromas.

**Figure 3:**
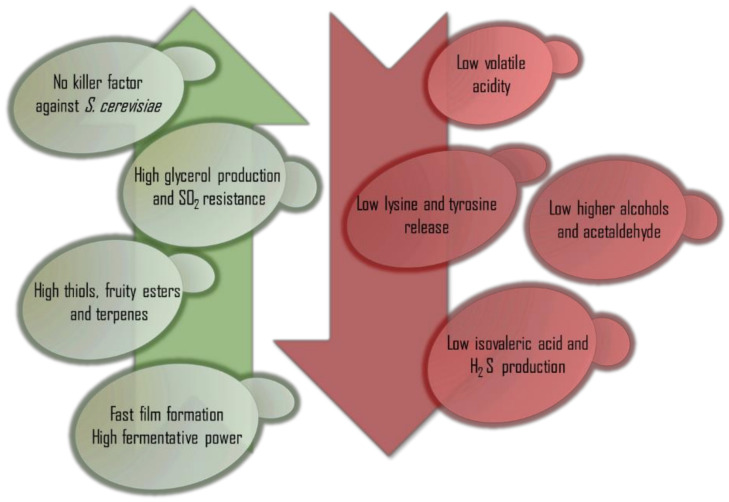
Summary of proposed *Pichia kluyveri* selection parameters.

The release of amino acids such as tyrosine and lysine, which are able to evolve to biogenic amines such as tyramine and cadaverine, must be reduced as much as possible during the selection process [73]. Figure 3 summarizes the proposed *P. kluyveri* selection parameters.

## 7. Conclusions

*P. kluyveri* improves wine quality parameters such as thiol, fruity ester, and terpene concentrations, mainly in sequential fermentation. Additionally, clear patterns such as lower ethanol and higher glycerol production, and higher **2**-phenyl ethyl acetate and lower hexanol production are observed in all research works. Other parameters such as malic acid and acetaldehyde reduction and methanol increase must be more deeply studied, as they are only reported once. Currently, only two strains are commercially available, so further strains should be selected to increase the market offerings. Possible occasional undesirable effects such as increased isovaleric acid, phenethylamine, H_2_S, or biogenic amine precursors, which seem to be strain-dependent, must be carefully studied in the future. Another technological factor of interest is the presence of killer toxins, which can enable the implantation of selected *S. saccharomyces* strains.

Due to its potential, *P. kluyveri* has generated interest in other fermentation products such as beer, cider, and cocoa in order to improve quality parameters related to sensory perception. Although *P. kluyveri* is the only *Pichia* species available on the yeast market, others such as *P. fermentans*, *P. guilliermondii*, *P. kudriavzevii*, *P. anomala*, and *P. membranifaciens* are being studied for winemaking purposes and new commercial strains could be available in the near future.

## Figures and Tables

**Table 1:** The main non-*Saccharomyces* yeast species of oenological importance and their influence on wine fermentation.

Species	Oenological Impact
*Torulospora delbrueckii*	Increased esters and thiols; acetic acid consumption [7,8]
*Lachancea thermotolerans*	Increased L-lactic acid, glycerol, and **2**-phenyl-ethanol [9,10,11]
*Metschnikowia pulcherrima*	Increased esters, terpenes, thiols, and aromatic complexity [12]
*Schizosaccharomyces pombe*	Increased deacidification by L-malic acid degradation [13]
*Candida zemplinina*	Increased glycerol and succinic acid; decreased acetic acid and higher alcohols [2]
*Hanseniaspora* spp.	Increased acetate esters and terpenes; biogenic amine adsorption [2]
*Hansenula anomala*	Low C6 alcohols; increased higher alcohols and acetate and ethyl esters [2]
*Zygosaccharomyces bailii*	Increased polysaccharides; acetic acid [2]
*Pichia guillermondii*	Increased color stability and 4-ethyl-phenol production [2]
*Pichia kluyveri*	Increased varietal thiols and esters [2]

**Table 2:** Main aroma compounds influenced by *P. kluyveri,* chemical structure, aromatic descriptor, and perception threshold.

Group	Aroma Compound	Structure	Odor Descriptor	Perception Threshold (ng/L)	Reference
Higher alcohols	**2**-methyl butanol	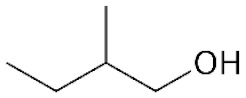	Harsh, nail polish remover	30,000	[36]
	**3**-methyl butanol	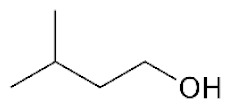	Harsh, nail polish remover	30,000	[36]
	**2**-phenyl ethanol	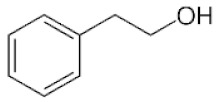	Rose	10,000	[36]
Esters	**2**-phenyl-ethyl acetate	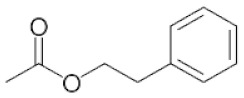	Rose, raspberry	250	[36]
	**2**-methyl-butyl acetate	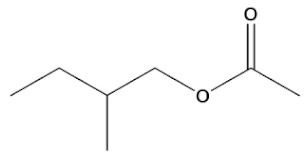	Banana	5	[36]
Terpenes	Linalool	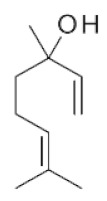	Flowery, fruity	6 for white varieties 15 for red varieties	[34]
	Hotrienol	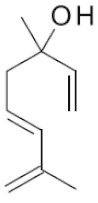	Faintly flowery, elderflower	110	[34]
Thiols	**3**-SHA	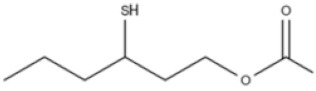	Passionfruit, box tree	4	[34]
	**3**-SH	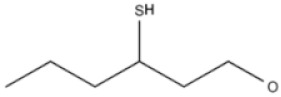	Grapefruit, citrus peel	60	[34]

## Data Availability

Not applicable.
